# Metabolic profiling of body fluids and multivariate data analysis

**DOI:** 10.1016/j.mex.2017.02.004

**Published:** 2017-02-20

**Authors:** Jean-Pierre Trezzi, Christian Jäger, Sara Galozzi, Katalin Barkovits, Katrin Marcus, Brit Mollenhauer, Karsten Hiller

**Affiliations:** a Luxembourg Centre for Systems Biomedicine, University of Luxembourg, Belvaux, Luxembourg; b Integrated BioBank of Luxembourg, Strassen, Luxembourg; c Functional Proteomics, Medizinisches Proteom-Center, Ruhr-University Bochum, Germany; d Paracelsus-Elena Klinik, Kassel, Germany; e University Medical Center Goettingen, Institute of Neuropathology and Department of Neurosurgery, Goettingen, Germany; f Braunschweig Integrated Centre of Systems Biology, University of Braunschweig, Rebenring 56, Braunschweig, Germany; g Department of Computational Biology of Infection Research, Helmholtz Centre for Infection Research, Braunschweig, Germany

**Keywords:** Metabolic profiling of body fluids and multivariate data analysis, Metabolomics, Metabolite extraction, Blood, Saliva, Cerebrospinal fluid, GC–MS, Data analysis, Body fluids

## Abstract

Metabolome analyses of body fluids are challenging due pre-analytical variations, such as pre-processing delay and temperature, and constant dynamical changes of biochemical processes within the samples. Therefore, proper sample handling starting from the time of collection up to the analysis is crucial to obtain high quality samples and reproducible results. A metabolomics analysis is divided into 4 main steps: 1) Sample collection, 2) Metabolite extraction, 3) Data acquisition and 4) Data analysis.

Here, we describe a protocol for gas chromatography coupled to mass spectrometry (GC–MS) based metabolic analysis for biological matrices, especially body fluids. This protocol can be applied on blood serum/plasma, saliva and cerebrospinal fluid (CSF) samples of humans and other vertebrates. It covers sample collection, sample pre-processing, metabolite extraction, GC–MS measurement and guidelines for the subsequent data analysis.

Advantages of this protocol include:

• Robust and reproducible metabolomics results, taking into account pre-analytical variations that may occur during the sampling process
• Small sample volume required
• Rapid and cost-effective processing of biological samples
• Logistic regression based determination of biomarker signatures for in-depth data analysis

## Graphical abstract

Quantitative multi-component analysis of body fluids with GC–MS. After sample collection, the samples are pre-processed and used for metabolite extraction. After this, GC–MS analysis and subsequent data processing and analysis are performed.

**Figure:**
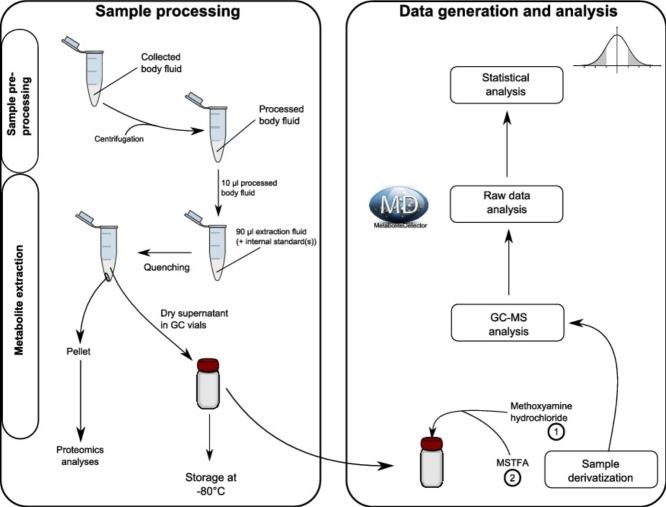

## Method details

### Materials

• 50 μL body fluid (for analysis in triplicates), such as plasma, serum, saliva or CSF (fresh or stored at −80 °C)
• Methanol (High purity, LC–MS grade) at −20 °C
• Internal standard [U-^13^C]Ribitol (Omicron Biochemicals, ALD-062)
• Sample collection tubes, such as sterile collection tubes (for CSF and saliva), EDTA and serum-separating tubes (for blood)
• Wet ice
• Methoxyamine hydrochloride 98% (Sigma-Aldrich, 226904)
• Pyridine 99.8% (Sigma-Aldrich, 270970)
• N-methyl-N-trimethylsilyl-trifluoroacetamide (Macherey-Nagel, 701270.110)
• Alkane standard mixture for performance tests of GC-systems (Sigma-Aldrich, 68281-10ml-F)
• GC glass vials with micro insert (gastight) 5–250 μL (various suppliers)
• (Magnetic) caps for GC glass vials (various suppliers)

All aqueous solutions used throughout this protocol should be prepared with MilliQ or deionized water (18.2 MΩ cm, <3 ppb TOC).

Note: The proper selection of the collection tubes is highly relevant for GC–MS based metabolomics analyses. In Section Choice of collection tubes we review and recommend several types of collection tubes for the different body fluid types. In general, we recommend the use of sterile tubes to reduce the risk of sample contamination.

### Equipment

• Reaction tube centrifuge, such as Eppendorf 5424R
• Refrigerated rotary vacuum evaporator, such as CentriVap Concentrator refrigerated (Labconco)
• Reaction tube shaker, such as Thermomixer comfort (Eppendorf)
• GC–MS instrument, such as Agilent 7890A GC System – Agilent 5975C inert XL MSD
• Autosampler, such as Gerstel Multi Purpose Sampler for automated derivatization and injection
• Agilent J&W DB-35MS, 30 m × 0.25 mm × 0.25 μm (L × I.D. × film thickness) + 5 m Duraguard or different brand

### Disclaimer

All protocols using biological material involving humans must be reviewed and approved by an ethical board and must be carried out in accordance with “The Code of Ethics of the World Medical Association” (Declaration of Helsinki).

### Choice of collection tubes

To get the best outcome, the choice of the correct sampling device for sample collection is crucial. Sampling devices may contain additives that are interacting with the analytes of interest and might even lead to non-reproducible results. In addition, not every collection device is suitable for all body fluids, and body fluid-specific collection devices must be used. We recommend using collection tubes without additives or containing additives (*e.g.* EDTA) that do not interfere with endogenous metabolite concentrations. For saliva and CSF the sampling can be done into sterile collection tubes without additives and stored at −80 °C after centrifugation.

For serum collection via venipuncture, plain collection tubes cannot be used. For serum, the blood is sampled into serum-separating tubes (SSTs) containing a gel separating the serum from blood cells. The clotting step is important and is usually done at RT for 30 min depending on the type of serum tube and the coagulation enhancer added. After centrifugation a physical barrier between serum and blood cells is formed. Serum can then be easily separated from the blood cells after centrifugation and used for metabolome analyses. Due to pre-analytical variations (*e.g.* temperature or pre-centrifugation delay), the metabolic profile can change very rapidly. Thermolabile and phosphorylated compounds are sensitive to pre-analytical variations (*e.g.* spontaneous biochemical reactions) which exacerbates metabolite analysis. It is therefore recommended to keep the samples at RT for the absolute necessary minimum period of time.

For plasma, anti-coagulating substances are used to prevent clotting of the blood sample. There are many options available, such as citrate and ethylenediaminetetraacetic acid (EDTA). In general, anticoagulants interfere with endogenous blood clotting processes by binding calcium ions from coagulation proteins. Thereby, clotting reactions are inhibited. We recommend using EDTA vacutainers as EDTA is not interfering with endogenous metabolites in contrast to other anti-coagulating substances such as citrate. Whereas citrate from collection tubes superimposes endogenous citrate, EDTA does not interfere with endogenous metabolites. Citrate and EDTA will be detected by GC–MS and may lead to a chromatographically overloaded peak causing analytical problems. However, this is not problematic under the described conditions due to chromatographic separation of these compounds and endogenous compounds.

The sampling procedure must be performed in a standardized manner to reduce variability emanating from circadian rhythm or other pre-analytical variations. In particular blood drawing shows a high variability during the course of the day. Thereby, it is crucial to standardize the sample collection procedures to specific day times and to monitor the exact sampling times for each donor. In general, blood collection should be performed after an overnight fasting of at least 8 h to reduce diet-related interactions in the metabolome.

### Sample pre-processing

Before metabolite extraction, the body fluid has to be separated from undesired biological material such as cells, debris or particles. Sample pre-processing is thereby separating the majority of the cells that would otherwise result in further biochemical reactions in a biological sample over time. Especially for blood it is essential to quickly remove all cellular material, in order to limit biochemical processes that emanate from the remaining cells.

Additionally, to avoid pre-analytical variations, the processing should be done within 1 h following the sample collection and the samples should be stored on ice (4 °C) during this period [1]. Pre-analytical variation, such as the storage temperature or the pre-centrifugation delay can highly impact the sample quality and should be standardized and monitored. The sample pre-processing procedures for the individual body fluids are as follows:

• For CSF samples, centrifuge the tubes at 2000 × *g*, 4 °C for 10 min [2].
• For serum, allow the samples to clot for 30 min at RT (in accordance with the collection tube manufacturer’s guide) and then centrifuge at 2000 × *g*, 4 °C for 10 min [3].
• For plasma, centrifuge the tubes at 2000 × *g*, 4 °C for 20 min [3].
• For saliva samples, centrifuge the tubes at 12,000 × *g*, 4 °C for 10 min. Due to the high mucus content and large food debris that can be present in freshly sampled saliva, we recommend a higher centrifugation speed as for the other body fluids [4] .

After sample pre-processing, the supernatant is transferred in new tubes. We recommend using cryotubes without additives in order to enable optimal storage conditions for metabolomics studies. The samples can either be used directly for the metabolite extraction or can be stored at −80 °C until further sample processing.

### Metabolite extraction

The samples should always be allowed to thaw at 4 °C that can either be done on wet ice or in cooling racks. The samples should be kept at low temperatures as biochemical reactions can occur at higher temperatures which significantly reduces sample quality. After thawing, the samples should be processed as quickly as possible to avoid changes in the sample quality due to pre-analytical variations, such as temperature and time.

The identical volume is removed three times from each sample and processed in parallel to assess the performance of the analytical procedure. The metabolomics standards initiative recommends favoring biological replicates over technical replicates [5]. However, in addition we recommend sample extraction in technical triplicates to better account for variation in the extraction and analysis process. An integral part of metabolite extraction is the quenching step. Quenching is the process by which all biochemical processes within the sample are suppressed. In addition, all proteins within the sample are precipitated. Within this protocol, this step is performed by a methanol water mixture.

The metabolite extraction procedures for the individual body fluids are as follows: The extraction fluid has to be prepared in advance and stored at −20 °C until metabolite extraction. Note: Additional internal standards can be used for monitoring of different substance classes. This protocol has been adapted from the plasma metabolite extraction and analysis protocol described by Jiye et al. [6].

Preparation of extraction fluid (8:1 MeOH/H_2_O ± IS mixture):

1. Prepare the internal standard solution (H_2_O + IS) by diluting [U^13^C]Ribitol with MilliQ water to obtain a concentration of 45 μg/mL
2. Mix 8 volumes of methanol (MeOH) with 1 volume of the internal standard solution (c([U^13^C]Ribitol) = 45 μg/mL) to obtain the final extraction fluid (pre-chilled at −20 °C) for metabolite extraction (c([U^13^C]Ribitol) = 5 μg/mL)

Metabolite extraction (in triplicate for each sample):

1. Mix 10 μL of either plasma, serum, CSF or saliva with 90 μL extraction fluid (−20 °C) in a 1.5-ml-reaction tube for quenching. We recommend to aliquot the extraction fluid before adding the samples and to keep all samples on ice.
2. Vortex thoroughly
3. Shake for 5 min on Thermomixer at 1400 rpm, 4 °C
4. Centrifuge for 5 min at 16,000 × g, 4 °C
5. Transfer 70 μL supernatant (contains metabolites) in GC vials with micro insert. Optionally, keep the pellet for protein extraction if applicable (see below)
6. Dry supernatant in a refrigerated rotary vacuum evaporator at −4 °C for a minimum of 1 h. Important: Make sure the samples are completely dry
7. Before taking out the vials, allow the refrigerated rotary vacuum evaporator to warm up to RT for 30 min to prevent water condensation in vials. This avoids problems during derivatization which is highly sensitive to humidity.
8. Tightly cap vials and store at −80 °C until GC–MS measurement

Optional extension to proteomics analyses:

After step 4, the supernatant contains polar metabolites and is used for subsequent metabolomics analysis. The pellet contains DNA, RNA and proteins, and can be applied for subsequent proteomics analysis. For this, reaction tubes are dried in a refrigerated rotary vacuum evaporator at −4 °C and stored at −80 °C until proteomics analysis.

### Derivatization and GC–MS analysis

Within this protocol, the GC–MS measurement includes a 2-step derivatization of the sample. Most of the metabolites present in body fluids contain polar functional groups, such as hydroxyl, carboxyl, thiol, phosphate or amine groups. Gas chromatography only separates gaseous compounds and therefore requires chemical derivatization to increase volatility of mostly polar metabolites.

In GC–MS-based metabolomics analyses, a 2-step derivatization is often applied by using methoxyamine hydrochloride and *N*-methyl-*N*-trimethylsilyl-triflouroacetamide (MSTFA) [7], [8]. In the first step, methoxyamine hydrochloride is used to reduce the chromatographic complexity of the samples, *e.g.* hexoses are fixed in open-chain form to avoid the detection of anomers. In the second step, silylation with MSTFA substitutes active protons of polar functional groups (*e.g.* hydroxyl groups) with trimethyl-silyl groups to increase metabolite volatility and metabolite stability.

We recommend using an automated sample derivatization to improve precision and accuracy of the derivatization step. After derivatization, the samples can be measured by GC–MS. The following GC–MS method protocol is optimized for the GC–MS measurement of the generated samples (see above):

#### Derivatization

Perform automated sample derivatization using an autosampler and sample preparation robot.

• Dissolve dried samples in 15 μL pyridine, containing 20 mg/mL methoxyamine hydrochloride
• Incubate at 40 °C for 90 min under shaking
• Add 15 μL *N*-methyl-*N*-trimethylsilyl-triflouroacetamide (MSTFA)
• Incubate at 40 °C for 30 min under continuous shaking

#### GC–MS analysis

GC–MS analysis is performed by using a gas chromatograph coupled to a mass spectrometer with a quadrupole analyzer and an electron ionization source, such as an Agilent 7890A GC coupled to an Agilent 5975C inert XL Mass Selective Detector (Agilent Technologies, Germany). The gas chromatograph is equipped with a 30 m DB-35MS capillary column + 5 m DuraGuard capillary in front of the analytical column (Agilent J&W GC Column). We recommend using a pre-column in front of the analytical column to preserve optimal chromatographic conditions. In addition, as (non-volatile) impurities can decrease chromatographic selectivity, a pre-column enables the preservation of the full length of the analytical column after column trimming.

The GC–MS measurement is performed in accordance to the following GC parameters (optimized for the before mentioned Agilent GC–MS system):

1. Split/Splitless inlet
  • Inlet temperature 270 °C
2. Split ratio 10:1
3. Injection volume: 1 μL
4. Carrier gas: Helium
5. Flow rate: 1.2 mL/min (constant)
6. GC oven
  • Temperature program: 90 °C for 1 min and increased to 320 °C at 15 °C/min, then held at that temperature for 8 min. Total run time for each sample is 24.3 min.
7. Mass selective detector (MSD) parameters
  • Temperature settings
    ○ Transfer line 280 °C
  • Ion source 230 °C
8. Quadrupole 150 °C
9. Electron ionization at 70 eV
10. Full scan mass spectra from m/z 70 to 700
11. Minimum scan rate: 4 scans/s

#### GC–MS raw data analysis

Deconvolution of mass spectra, peak picking, integration, and retention index calibration are performed using the MetaboliteDetector software Version 2.5 or higher [9]. Compounds are identified using a mass spectral reference library.

The following deconvolution settings are applied:

• Peak threshold: 5
• Minimum peak height: 5
• Bins per scan: 10
• Deconvolution width: 5 scans
• No baseline adjustment
• Minimum 15 peaks per spectrum
• No minimum required base peak intensity

Retention index calibration is based on a C10–C40 even n-alkane mixture. For detailed information on the use of the different settings, please visit http://md.tu-bs.de/.

### GC–MS quality assurance

**Fig. 1:**
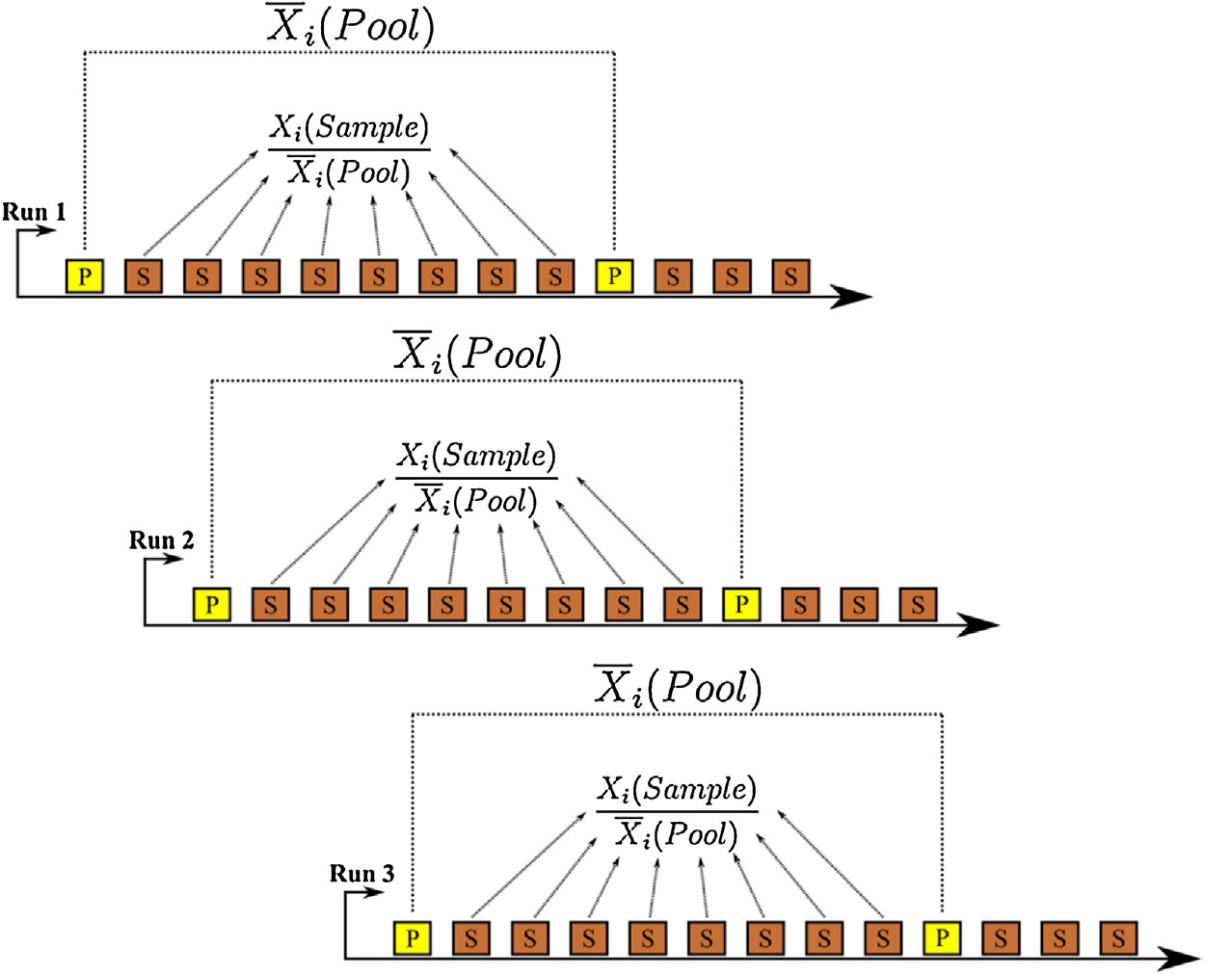
Normalization by reference pool measurements. In a GC–MS sequence plan, every 8th position a reference pool sample is measured. For pool normalization, the average of the 2 nearest pools is calculated for each metabolite *i* ($\bar{X_{i}}$). Then, for each sample the metabolite intensity of each metabolite *i* is divided by the corresponding average $\bar{X_{i}}$ of the pools: $\frac{X_{i}\left(Sample\right)}{{\bar{X}}_{i}\left(Pool\right)}$.

To increase the quality of the GC–MS measurement, the following points should be carefully considered:

1. Extraction blanks. Extraction blanks are blanks for which the metabolite extraction procedure was followed by using MilliQ water instead of the sample of interest. These blanks take into consideration contaminations (in *e.g.* solvents, derivatization reagents) and other problems that might occur during the extraction or the derivatization step and should therefore always be monitored. In addition, extraction blank information can be used for baseline substraction.

2. Internal Standards. Internal standards are ideally stable isotopically labelled compounds that should be introduced in the sample processing as early as possible, (*e.g.* in the metabolite extraction fluid), and can be easily distinguished from endogenous metabolites by mass spectrometry. Ideally, internal standards have the same substance class as the compounds present in the sample to be analyzed and can therefore be used to correct for uncontrolled sample losses or compound degradation and subsequent sample losses, to improve method precision and accuracy. As the whole metabolite extraction and measurement procedure is performed with these internal standards, a monitoring of losses due to various error sources is achieved.

3. Quality control (QC) samples. For a given experiment, a small proportion of each sample can be used to form a pool by mixing equal amounts within the experiment. The QC sample is then extracted and inserted in the measurement sequence multiple times. We recommend extracting sufficient QC samples so that every 8th sample of the GC–MS run sequence is a pool sample. QC samples do not only enable a monitoring of the GC–MS measurement quality, such as instrument drifts/sensitivity drops and chromatographic changes (*e.g.* retention time shifts) but can also be used for data normalization [10]. In this case, normalization is done for each metabolite by dividing the sample metabolite intensity by the average of the chronologically (within the sequence) nearest pool sample metabolite intensities (Fig. 1). The main advantage of QC samples is that they contain the average of all metabolites within all the samples that are analyzed which enables the monitoring of the measurement quality for each individual metabolite even in non-targeted mode and for long sequences. These QC samples serve as data normalization tool for untargeted metabolomics approaches to remove analytical variation (see Section Statistical analysis) [11]. This is in contrast to the normalization by internal standards which is specific to the chemical classes similar to the internal standards. Thereby, normalization by internal standards shows a better performance in a targeted metabolomics analysis [12]. In this protocol, we describe an untargeted metabolomics method and thereby use QC normalization.

4. GC–MS sequence plan. A carefully planned measurement sequence should be set up for the GC–MS run involving the samples, pools and blanks. We recommend to first start with the measurement of an alkane mix that can be used for further RI calibration, followed by a clean run (only injecting MSTFA) without derivatization. Second, a blank should run followed by the samples. To equilibrate the GC column for the matrix, 2–3 pool samples should be measured. We also recommend that every 8th measurement is a pool sample. The samples should also be randomized within the sequence and not follow a pre-defined scheme which is very important for time-resolved experiments.

Sensitivity drop emanating from a prolonged measurement (>180 samples) and complex samples can occur. Therefore, instrument performance and robustness must be checked on regular intervals during measurements. The performance check should ideally be performed by the monitoring of the QC samples. In case of a sensitivity drop for the metabolites of interest within the QC samples, further maintenance steps are required (e.g. column trimming, liner replacement).

### Statistical analysis

After deconvolution and quantification, the processed metabolomics data can be directly used for statistical analysis with commercial or open-source software. The most important step of a statistical analysis is data normalization to remove unwanted analytical variation and/or to correct for inter- and intrabatch variability.

In this protocol, the metabolomics data is normalized by the generated reference pools (QC samples) that have been measured at every 8th position during the GC–MS run. To normalize by pools, the following procedure has to be followed for each individual metabolite (Fig. 1):

1. Calculate the average of the metabolite intensities of the chronologically 2 nearest pools
2. Divide the metabolite intensity of the sample of interest by the calculated average

Before statistical analysis, the GC–MS measurement and metabolite extraction should be evaluated by internal standards. Especially over long sequences, a decrease in sensitivity can be observed over time. To monitor the decrease of sensitivity, internal standard signals can be observed over time and a decision about the measurement quality can be made. In addition, samples that have not been properly extracted can be identified as outliers by internal standard monitoring.

Whereas classical ANOVA or *t*-test methods are sufficient for simple statistical comparisons, more sophisticated methods are required to adequately analyse the very large amounts of data generated by metabolomics technologies. A review of the use of machine learning algorithms in metabolomics has recently been published [13].

In this protocol, we provide an example of a supervised machine learning algorithm based on logistic regression for sample classification. The normalized data should be partitioned in training and test set. The training set is used for the calculation of the model parameters by maximum likelihood estimation and the test set is used for the evaluation of the model performance. The model performance can be evaluated by receiver operating characteristics (ROC) curves enabling the calculation of an optimal decision threshold for unknown sample identity predictions and the respective specificity and sensitivity. After feature selection and model parameter calculation, the logistic regression model can be used to predict the identities of unknown samples. The results are stated as probabilities. As the learning algorithm tends to model noise instead of the desired information, it is highly recommended to test the modeling process for overfitting. To avoid overfitting, cross-validation and/or regularization of the model process are required [14].

A detailed description of the modelling process and the corresponding R script written under R version 3.2.2. using the “pROC” package [15] and example files can be found in supplementary data (Supplementary data 1–3).
